# Supplementary figures and images for: Chronic stress impairs autoinhibition in neurons of the locus coeruleus to increase asparagine endopeptidase activity
Source: eLife. 2025 Oct 9;14:RP106362. doi: 10.7554/eLife.106362 (PMC12510684; doi:10.7554/eLife.106362)

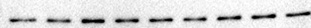

Supplement: Figure 3—source data 1. [file elife-106362-fig3-data1.zip › Figure 3C alpha2A original file.jpg]

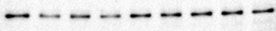

Supplement: Figure 3—source data 1. [file elife-106362-fig3-data1.zip › Figure 3C GAPDH original file.jpg]

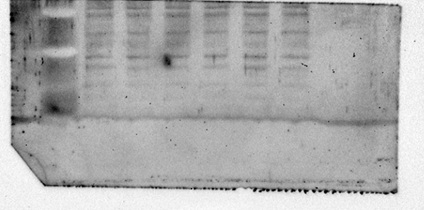

Supplement: Figure 3—source data 1. [file elife-106362-fig3-data1.zip › Figure 3F alpha2A original file.jpg]

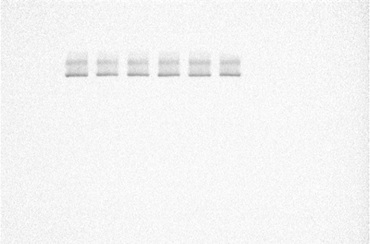

Supplement: Figure 3—source data 1. [file elife-106362-fig3-data1.zip › Figure 3F Na-K-ATPase original file.jpg]

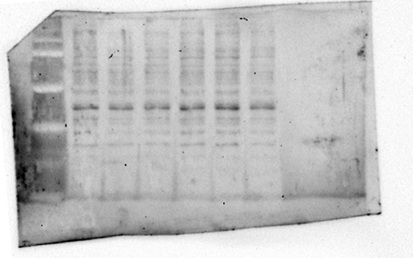

Supplement: Figure 3—source data 1. [file elife-106362-fig3-data1.zip › Figure 3G alpha 2A original file.jpg]

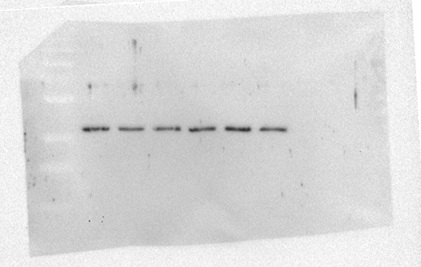

Supplement: Figure 3—source data 1. [file elife-106362-fig3-data1.zip › Figure 3G beta-actin original file.jpg]

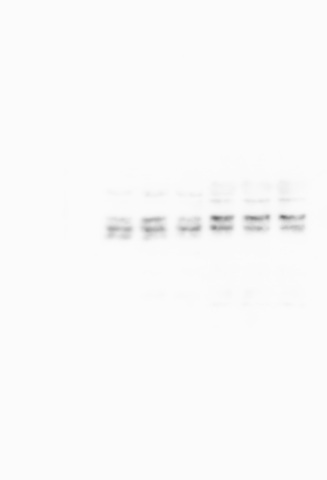

Supplement: Figure 5—source data 1. [file elife-106362-fig5-data1.zip › Figure 5G tauN368 original file.jpg]

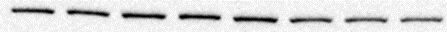

Supplement: Figure 5—source data 1. [file elife-106362-fig5-data1.zip › Figure 5A beta-actin 1 original file.bmp]

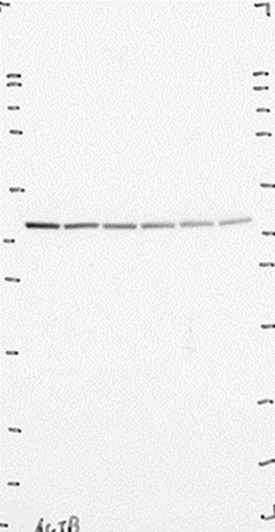

Supplement: Figure 5—source data 1. [file elife-106362-fig5-data1.zip › Figure 5A beta-actin 2 original file.jpg]

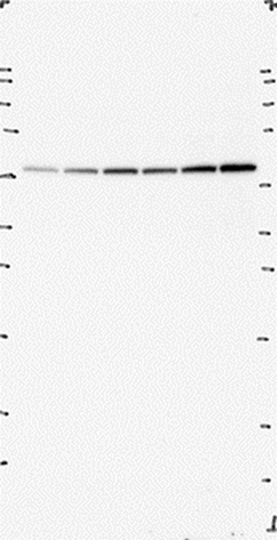

Supplement: Figure 5—source data 1. [file elife-106362-fig5-data1.zip › Figure 5A MAO-A original file.jpg]

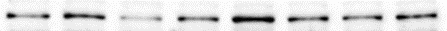

Supplement: Figure 5—source data 1. [file elife-106362-fig5-data1.zip › Figure 5A TH original file.bmp]

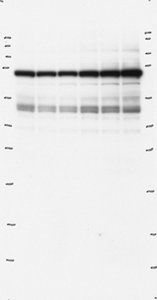

Supplement: Figure 5—source data 1. [file elife-106362-fig5-data1.zip › Figure 5D AEP original file.png]

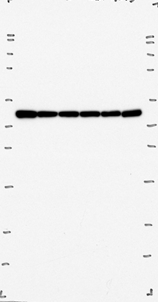

Supplement: Figure 5—source data 1. [file elife-106362-fig5-data1.zip › Figure 5D beta-actin original file.png]

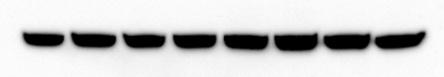

Supplement: Figure 5—source data 1. [file elife-106362-fig5-data1.zip › Figure 5G beta-actin 1 original file.jpg]

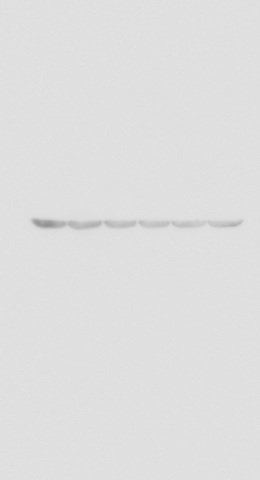

Supplement: Figure 5—source data 1. [file elife-106362-fig5-data1.zip › Figure 5G beta-actin 2 original file.jpg]

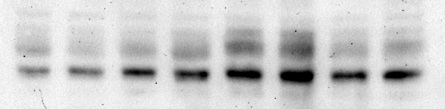

Supplement: Figure 5—source data 1. [file elife-106362-fig5-data1.zip › Figure 5G tau original file.jpg]
