# Supplementary material for: Chronic stress impairs autoinhibition in neurons of the locus coeruleus to increase asparagine endopeptidase activity
Source: eLife. 2025 Oct 9;14:RP106362. doi: 10.7554/eLife.106362 (PMC12510684; doi:10.7554/eLife.106362)
Supplement: Figure 3—source data 2. [file elife-106362-fig3-data2.pdf]

Figure 3

C

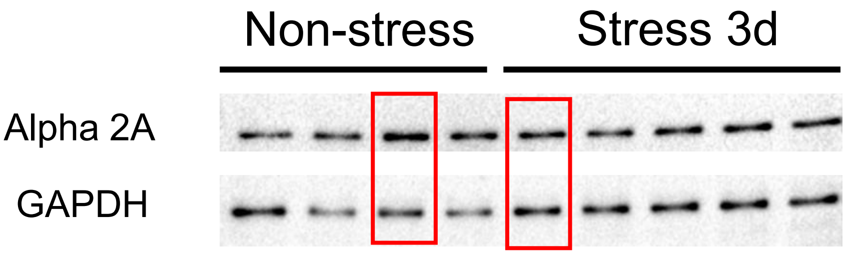

The highlighted images are shown in the figure.

F

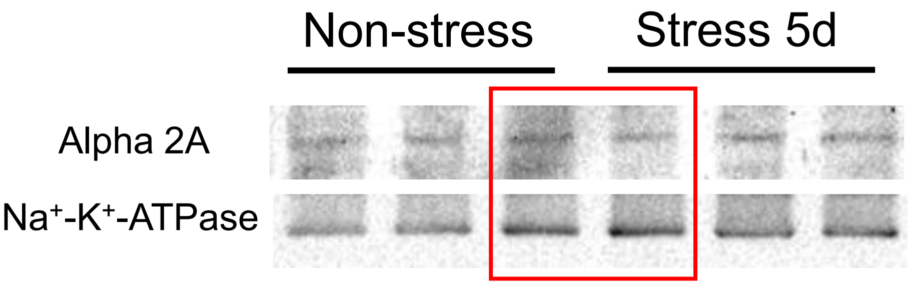

The highlighted images are shown in the figure.

G

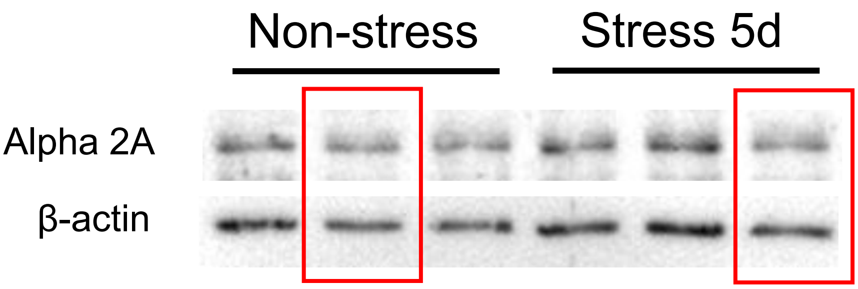

The highlighted images are shown in the figure.
